# Mr. Peck's Observations on the Leech Worm

**Published:** 1810-03

**Authors:** 


### MR. PECK'S OBSERVATIONS ON THE LEECH WORM.

### *Changes that I have observed in the Animal before any particular Alteration of the Weather.*

1. WHEN the leech lies motionless at the bottom of the glass, and is frequently in a spiral form, the weather in summer will be serene and beautiful: the same denotes clear frosty weather in winter.

2d. If

2. If it creeps up to the top of its lodging, it will rain within twenty-four hours in summer, and snow in winter.

3. When the leech gallops through its limpid habitation with swiftness, it denotes wind, and seldom rests until it blows hard.

4. When the leech lodges almost constantly out of the water, and discovers uncommon uneasines in violent throes and convulsive-like motions, a storm of thunder and rain will succeed.

*Method of keeping Leeches.*

1. Put a few into an eight-ounce phial two-thirds full of spring water, with some fine sand or moss at the bottom. As the leeches have no other evacuation but through the pores of the skin, which passes from them in perspirable matter, and adheres to the body in the state of slime, which, if not timely removed, prevents these evacuations, and causes the death of the worm; the use of sand, or moss, is, that it may rub the slime off its body, which afterwards floats in the water. Over the top of the phial tie a piece of leather pricked full of holes to admit air.

2. The water must be changed once a week: spring water is the best. Sometimes it is necessary, when there is a great change of temperature between the water and that contained in the phial, only to put half or two-thirds of the fresh to the other. Leeches should be kept in a cool situation in summer and a rather warm one in winter.

3. The leeches that have been used for bleeding should be kept in a separate phial till they appear perfectly well.

*Directions for using Leeches in Bleeding.*

1. It is necessary to clean the skin from any foreign matter * that may have been applied or adheres to it, with soap and water: afterwards rub it dry with a clean cloth, as liniments, &c. which are frequently applied in cases of bruises, or sprains, prevent them from taking hold, and if any do so, they die: any part where hair grows must be clean shaved, to prevent the hair from annoying them. These are precautions that are necessary.

2. When leeches are applied, the patient should be in as horizontal a position as possible. Then take a wine or any other glass large enough to give room for the quantity that it is wished to take hold at once, being much better

* Such as the limentum saponis, solutio ammoniæ volatilis, &c.

than the fingers; it gives the worms free motion in their circumscribed limits, retains them in their proper place and supports them from falling. The glass should be reclined on one side to admit a free access of air. The leeches should be chosen large, to answer their purposes the more effectually. When they seem sufficiently filled, a small portion of salt should be put to their mouths, which will cause them to fall off, being better than taking them with the fingers, as it bruises them.

*Treatment of the Leeches after they are satiated with Blood.*

Place the leech on a clean plate; take a little common salt rubbed fine, about the size of a pinch of snuff, and place it in contact with the mouth of the worm; it will remain a short time in a state of torpor, after which it will disgorge part of the blood; a little more salt may then be placed near its mouth, repeating it until it is all disgorged, taking care that no part of the salt touch any other part of its body, which blisters, and is frequently the death of the leech. When the worm returns to its natural size, it may then be put into a bason of water; if it has received no injury it will frisk about and appear lively, if sickly, it will sink to the bottom: should this be the case, place it in a separate phial till well. Every leech should have a clean plate to disgorge itself on.

These observations have occurred in practice; and I am convinced that if they are strictly attended to, the mortality amongst leeches will be much lessened.

N. B. Those who wish to use a leech as a weather-glass, should choose one that has not been used for bleeding; for after they have been used they are frequently sickly, and will bury themselves in the sand for days together.

